# Device therapy for patients with atrial fibrillation and heart failure with preserved ejection fraction

**DOI:** 10.1007/s10741-023-10366-7

**Published:** 2023-11-08

**Authors:** Zixi Zhang, Yichao Xiao, Yongguo Dai, Qiuzhen Lin, Qiming Liu

**Affiliations:** 1 Department of Cardiovascular Medicine, The Second Xiangya Hospital, Central South University, Changsha, 410011 Hunan Province People’s Republic of China; 2 Department of Pharmacology, Wuhan University TaiKang Medical School (School of Basic Medical Sciences), Wuhan, 430071 Hubei Province People’s Republic of China

**Keywords:** Atrial fibrillation (AF), Heart failure with preserved ejection fraction (HFpEF), Cardiac resynchronization therapy (CRT), Cardiac contractility modulation (CCM), Vagus nerve stimulation (VNS), Left ventricular expanders (LVEs)

## Abstract

Device therapy is a nonpharmacological approach that presents a crucial advancement for managing patients with atrial fibrillation (AF) and heart failure with preserved ejection fraction (HFpEF). This review investigated the impact of device-based interventions and emphasized their potential for optimizing treatment for this complex patient demographic. Cardiac resynchronization therapy, augmented by atrioventricular node ablation with His-bundle pacing or left bundle-branch pacing, is effective for enhancing cardiac function and establishing atrioventricular synchrony. Cardiac contractility modulation and vagus nerve stimulation represent novel strategies for increasing myocardial contractility and adjusting the autonomic balance. Left ventricular expanders have demonstrated short-term benefits in HFpEF patients but require more investigation for long-term effectiveness and safety, especially in patients with AF. Research gaps regarding complications arising from left ventricular expander implantation need to be addressed. Device-based therapies for heart valve diseases, such as transcatheter aortic valve replacement and transcatheter edge-to-edge repair, show promise for patients with AF and HFpEF, particularly those with mitral or tricuspid regurgitation. Clinical evaluations show that these device therapies lessen AF occurrence, improve exercise tolerance, and boost left ventricular diastolic function. However, additional studies are required to perfect patient selection criteria and ascertain the long-term effectiveness and safety of these interventions. Our review underscores the significant potential of device therapy for improving the outcomes and quality of life for patients with AF and HFpEF.

## Introduction

Atrial fibrillation (AF) is a rapid supraventricular arrhythmia characterized by irregular electrical activity and ineffective atrial contractions. The incidence of AF gradually increases with age and has become a major public health concern [1]. Currently, heart failure (HF) is recognized as the most common complication in patients with AF, who have a fourfold higher risk of death from HF than from stroke [2]. Furthermore, AF is the most common type of arrhythmia in patients with HF, occurring in 24 − 44% of patients with acute HF and 33% of those with chronic HF [3]. The correlation between AF and heart failure with preserved ejection fraction (HFpEF) is more pronounced than that between AF and heart failure with reduced ejection fraction (HFrEF) [4, 5]. The incidence of HFpEF in patients with AF is approximately five times higher than that in patients without AF, and women generally have a higher incidence of HFpEF than men [6, 7]. Over 30% of patients with HFpEF experience concomitant AF [8]. The DOSE (Diuretics Optimization Strategies Evaluation) study [9] revealed that in patients with acutely decompensated HFpEF, AF can be prevalent in up to 69% of cases. Furthermore, existing evidence suggests that the presence of AF in HFpEF increases the risk of all-cause mortality and stroke, particularly when AF is incident [10]. These findings indicate a potential interplay between AF and HFpEF, leading to the formation of a vicious cycle.

Considering the observed clinical comorbidity between AF and HFpEF, shared pathophysiological mechanisms likely underlie both conditions. Both AF and HFpEF share common risk factors and comorbidities, such as aging, hypertension, obesity, and sleep apnea [1, 11]. This elevates the risk of developing both conditions. Current studies indicate that specific proinflammatory cytokines, such as tumor necrosis factor, interleukin-1, and interleukin-6, play roles in the pathogenesis of HFpEF [12], suggesting that HFpEF might be an inflammatory disorder. This proinflammatory environment in HFpEF can lead to endothelial dysfunction, oxidative stress, microvascular inflammation, and chronic fibrotic changes [13]. These factors contribute to diastolic dysfunction, which is also a pivotal mechanism in the development and persistence of AF. HFpEF can lead to left atrial enlargement and increased atrial fibrosis, disrupting gap junction distribution and intercellular coupling in fibrotic areas [14, 15]. This contributes to electrical remodeling, fostering the onset of AF. Additionally, HFpEF may elevate the activity of adrenergic and renin-angiotensin-aldosterone systems, promoting atrial fibrosis and AF development [16].

AF itself can lead to atrial dilation, atrial fibrosis, and impaired atrial function, thereby promoting the occurrence of HFpEF [17]. Remodeling of the atrioventricular annular associated with AF, along with the progressive development of mitral and tricuspid regurgitation (TR), may also represent another mechanism for HFpEF [18]. In patients with persistent AF, depletion of atrial natriuretic peptide can facilitate vasoconstriction and edema, providing a potential foundation for the development of HFpEF [19]. Additionally, AF is associated with left ventricular myocardial fibrosis, which contributes to diastolic dysfunction and HFpEF [20].

Currently, novel guideline-directed medical therapy (GDMT), including angiotensin receptor-neprilysin inhibitors, beta-blockers, mineralocorticoid receptor antagonists, and sodium-glucose cotransporter-2 inhibitors, has emerged as the preferred treatment approach for HF [21, 22]. Landmark trials have unequivocally shown significant benefits of this therapeutic regimen in patients with HFrEF [23–25]. Historically, certain medications such as sodium-glucose co-transporter-2 inhibitors and angiotensin receptor-neprilysin inhibitors have been spotlighted for their potential effectiveness in HFpEF patients [26–31]. However, emerging data from the STEP-HFpEF trial underscores the notable advantages of glucagon-like peptide-1 receptor agonists for obese HFpEF patients, especially in enhancing their quality of life [32]. It is worth noting that despite these advancements, drug treatments for HFpEF still face challenges in efficacy, and a definitive treatment strategy for AF patients complicated by HFpEF remains elusive. This article reviews the latest developments in mechanical treatments for AF and HFpEF and aims to advance the standardized management of this patient population.

## Clinical features

**Fig. 1:**
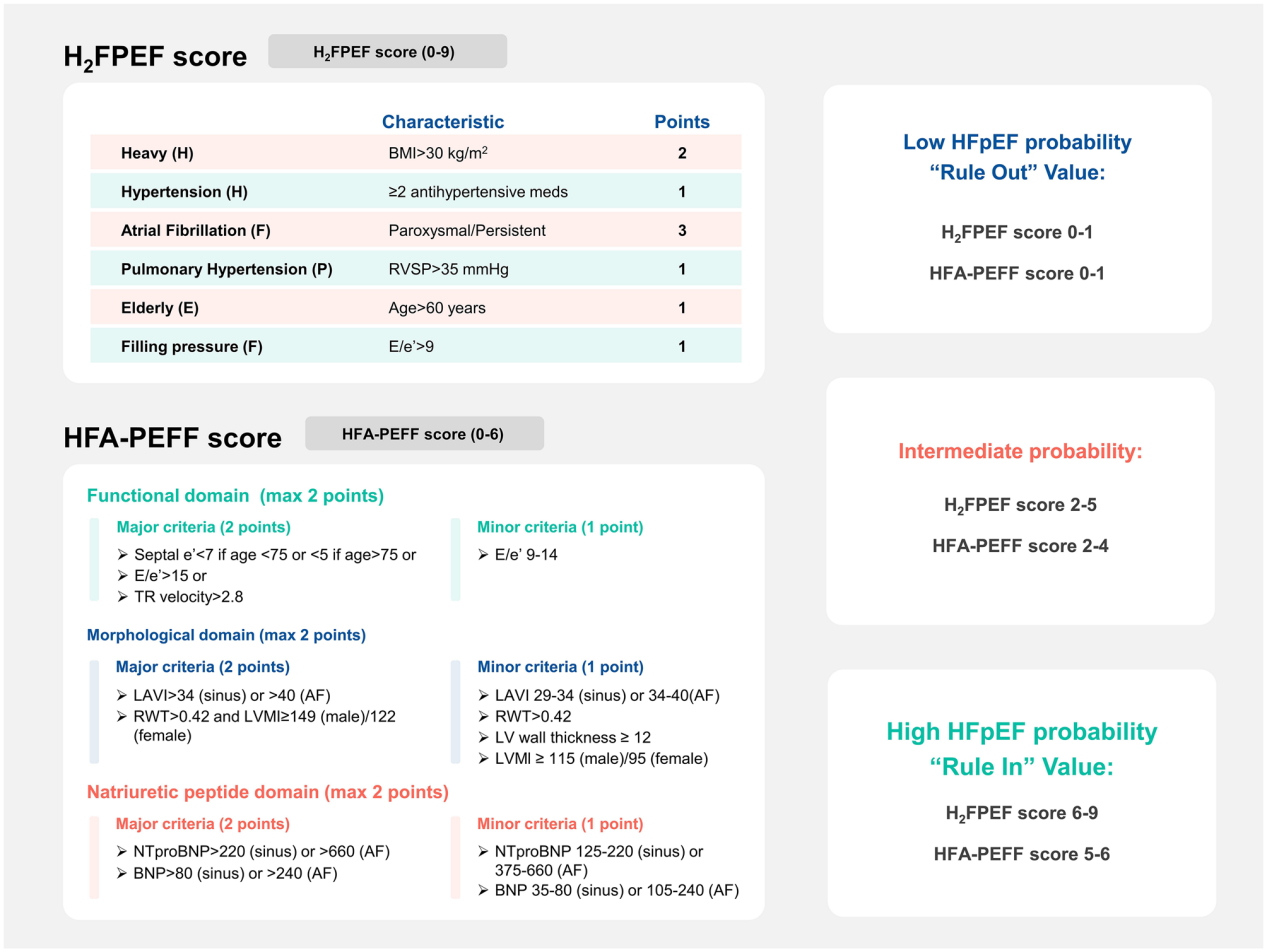
H2FPEF score and HFA-PEFF score. BMI, body mass index; RVSP, right ventricular systolic pressure; e’, septal mitral annulus tissue relaxation velocity in early diastole; E/e’, the ratio of early diastolic mitral inflow velocity to septal mitral annulus tissue relaxation velocity; TR, tricuspid regurgitation; LAVI, left atrial volume index; RWT, relative wall thickness; LVMI, left ventricular mass index; BNP, B-type natriuretic peptide; NT-proBNP, N-terminal pro-B-type natriuretic peptide; AF, atrial fibrillation; LV, left ventricle; HFpEF, heart failure with preserved ejection fraction

Both AF and HFpEF may present with symptoms such as palpitations, chest tightness, and dyspnea. B-type natriuretic peptide (BNP) levels are increased during AF episodes and rapidly normalize after conversion to sinus rhythm [33]. Diagnosing AF complicated by HFpEF based on clinical presentation and BNP levels alone may be challenging. Diagnosing HFpEF is straightforward in cases of volume overload. However, AF with a capacity imbalance may impact the assessment of left ventricular ejection fraction (LVEF), which may lead to an incorrect HFpEF diagnosis. Therefore, the diagnostic thresholds of BNP and NT-proBNP for AF complicated by HFpEF should be increased to 240 pg/mL and 660 pg/mL, respectively [3]. In addition to traditional diagnostic methods, the H2FPEF score and HFA-PEFF score can be used to diagnose HFpEF (Fig. 1). The study by Sepehrvand et al. [34] found that an H2FPEF score of > 2 had a sensitivity of 89–90% to detect HFpEF and that an H2FPEF score < 6 had a specificity of 82% to rule out HFpEF in the Alberta HEART population. However, it should be noted that the population recruited into Alberta HEART is nonrandom, and differences in HFpEF prevalence will influence positive and negative predictive values. Additionally, patients with higher H2FPEF scores are at a higher risk of adverse events [34]. The HFA-PEFF score is the other new diagnostic algorithm that accounts for various factors, and higher scores are associated with an increased risk of rehospitalization and all-cause mortality in patients with HF [35]. Although specificity was robust for both scores, sensitivity was poorer for HFA-PEFF, with a false-negative rate of 55% for low-probability scores compared with 25% using the H2FPEF score [36]. These scores offer new methods for diagnosing AF and HFpEF, but they are not without limitations. For instance, the H2FPEF score overlooks BNP levels, and its accuracy and utility require thorough evaluation. Furthermore, the HFA-PEFF score is overly lengthy and complicated, challenging its widespread implementation.

In the era of big data, artificial intelligence technology has become increasingly sophisticated, and intelligent algorithms based on machine learning have resulted in breakthroughs in the medical field [37]. These breakthroughs may be used to develop important methods of auxiliary diagnosis and treatment in the future. Currently, deep learning models based on echocardiography have been used to classify the degree of diastolic dysfunction and aid in the diagnosis of HFpEF [38]. These models can identify HFpEF phenotypes with different clinical features and long-term prognoses [39], providing effective evidence for the stratified diagnosis and treatment of HFpEF. Additionally, machine learning based on different biomarkers can identify HFpEF subgroups with different biomarker spectra [40], which may reveal different underlying pathological and physiological pathways of HFpEF.

## Challenges in pharmacological and interventional treatments

The efficacy of current medications for managing AF and HFpEF often falls short, leading to recurring symptoms or disease progression. Adverse effects from these medications, ranging from dizziness to increased bleeding risk, can affect patient adherence and overall well-being [41]. With AF and HFpEF patients frequently having multiple comorbidities, polypharmacy becomes a concern, introducing potential drug interactions and complicating treatment [42–44]. 

Despite the declining mortality rate in HFrEF patients due to new anti-HF drugs, the mortality rate in HFpEF patients remains concerning [45, 46]. This underscores the need for alternative treatments for AF and HFpEF [47, 48]. Ablation therapy, especially pulmonary vein isolation (PVI), has emerged as a significant treatment for AF [49]. Radiofrequency ablation (RFA) is more effective than drug therapy for maintaining sinus rhythm, reducing readmission, and improving diastolic function [50, 51]. In a detailed analysis of the CABANA trial data (trial code NCT00911508), Packer et al. [52] ascertained that catheter ablation outperformed antiarrhythmic drugs in enhancing the quality of life and curtailing AF recurrence specifically among patients with coexisting AF and HFpEF.

**Fig. 2:**
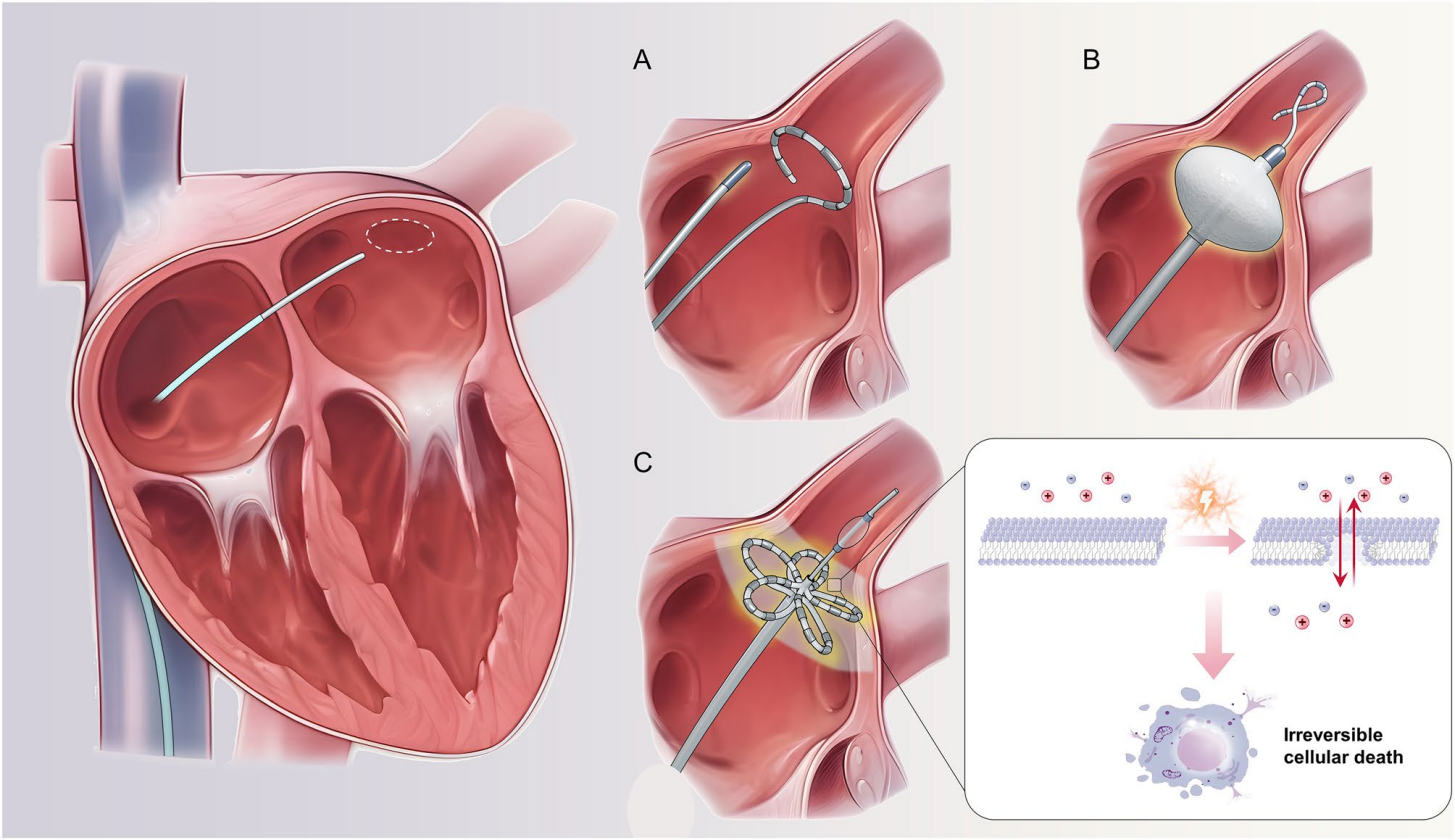
Diagrams of radiofrequency catheter ablation, cryoballoon ablation, and pulsed-field ablation. The image displays three distinct techniques for ablation. **A** Radiofrequency catheter ablation, which effectively achieves PVI through thermal ablation. **B** Balloon ablation in which cryogenic balloons are used to isolate the pulmonary vein. **C** Pulsed-field ablation in which irreversible micropores are formed in the myocardial membrane by administering pulsed electric fields. These micropores enable various ions to penetrate the membrane, ultimately disrupting the vital internal environment of cardiomyocytes and leading to their death. This process eventually results in the successful isolation of the pulmonary vein. The inset image in (**C**) provides a visual representation of how pulsed-field ablation functions

While RFA has proven effective, newer ablation technologies such as cryoballoon ablation and pulsed field ablation (PFA) offer potential advantages (Fig. 2). These methods promise safety and efficiency, with PFA notably preserving surrounding cardiac structures [53]. However, comprehensive research on these techniques is still in its infancy, warranting further exploration for their efficacy and safety in AF and HFpEF patients.

## Instrumental therapy and management

In the evolving landscape of cardiovascular care, the management of patients presenting with both AF and HFpEF has emerged as a multifaceted challenge. In this context, instrument-based interventions have garnered significant attention and promise. This comprehensive exploration delves into the realm of device therapies. By examining these interventions, we aim to shed light on their potential roles in improving the clinical outcomes and quality of life of patients with AF and HFpEF.

### Cardiac resynchronization therapy

Cardiac resynchronization therapy (CRT) achieves biventricular pacing (BVP) by increasing left ventricular pacing, which significantly improves heart function and increases long-term patient survival rates [54, 55]. There is currently limited evidence supporting the significant improvement of HFpEF prognosis after CRT; however, its potential lies in its ability to improve systolic and diastolic dyssynchrony and provide chronotropic support, which may increase the diastolic filling time of the heart [55–57]. A case report in 2010 showed that a woman with HFpEF and left bundle-branch block (LBBB) experienced improved clinical symptoms after receiving CRT [58], suggesting that CRT may benefit specific populations with HFpEF.

BVP-mode CRT significantly improves the prognosis of patients with HF; however, one-third of patients do not respond to CRT [59, 60]. BVP is not equivalent to physiological pacing but rather to fusion pacing formed by two ectopic pacing points. The risk of HF hospitalization and AF incidence significantly increases when the cumulative percentage ventricular pacing burden is above a threshold of 40% [61, 62]. Therefore, achieving greater ventricular resynchronization in the form of physiological pacing can significantly improve heart function and avoid pacemaker-induced cardiomyopathy [62, 63].

The His bundle is an extension of the physiological structure of the atrioventricular node that is connected to the left and right bundle branches and participates in the formation of the ventricular conduction system. Therefore, HBP is a new pacing mode that can replace BVP. Studies have shown that HBP-mode CRT can improve cardiac function in patients with AF and HFpEF after atrioventricular node ablation and is beneficial for antiventricular remodeling [64]. However, HBP has several limitations in clinical practice, including high thresholds and low sensing, owing to blocking characteristics similar to that of the atrioventricular node. Additionally, implantation difficulty and low surgical success rates are common challenges for HBP [65]. Almost half of the patients with HF combined with LBBB cannot normalize their QRS waves after HBP, and the benefits of HBP are limited for patients with intraventricular conduction block [66]. The concept of LBBP has been developed to address these issues [67]. LBBP avoids the area of the conduction block and captures the main trunk and proximal branches of the left bundle branch at their more distal end. Moreover, LBBP has lower operation difficulty and requires lower precision in lead placement, allowing it to be the optimal pacing mode for patients with LBBB [68, 69]. LBBP combined with atrioventricular node ablation can achieve clinical benefits similar to those obtained with HBP in patients with AF, with lower sensing thresholds and higher success rates. This has been recognized as a safe and effective treatment option. [70]

Currently, there is a lack of high-level clinical evidence directly demonstrating the significant benefits of CRT in patients with AF and HFpEF. Furthermore, current guidelines do not recommend CRT for patients with HFpEF [54]. However, CRT with HBP or LBBP in combination with atrioventricular node ablation may be a feasible alternative option for patients with coexisting LBBB or significant ventricular dyssynchrony with limited response to conventional treatments.

### Cardiac contractility modulation

Cardiac contractility modulation (CCM) is an innovative implantable electronic device used to treat chronic HF. The device features a pulse generator, which functions similar to a pacemaker, and two active fixation leads that are typically placed in the right ventricular septum with an interelectrode spacing of at least 2 cm. The device delivers a nonexcitatory biphasic signal of 7.5 V and 20 ms to the right ventricular septum during the absolute refractory period, promoting phosphorylation of phospholamban in the patient’s sarcoplasmic reticulum. Phosphorylated phospholamban dissociates from Ca^2+^-ATPase 2a on the sarcoplasmic reticulum, leading to increased intracellular Ca^2+^ concentration and a positive inotropic effect without increasing myocardial oxygen consumption, which improves cardiac function [71, 72].

Long-term CCM use can significantly enhance exercise tolerance and patient quality of life. According to the FIX-HF study (NCT01381172), CCM implantation dramatically increased LVEF, 6-min walking distance, and peak oxygen consumption and markedly reduced the risks of cardiovascular death and hospitalization for HF. The overall treatment effectiveness of CCM was found to be over 90% [73].

CCM has been approved for patients with symptomatic HFrEF with normal or slightly prolonged QRS duration in the European Union, China, India, Brazil, and other countries [74]. CCM has demonstrated significant benefits, particularly in patients with a baseline LVEF between 35 and 45%. At the molecular level, CCM improves calcium regulation in patients with HFrEF, reverses the fetal gene program associated with HF, and improves cardiac remodeling [74]. However, there is limited clinical evidence for the use of CCM in patients with HFpEF. In 2016, a case report described the implantation of CCM in two 59-year-old women with HFpEF. The patients showed improvements in cardiac function, 6-min walk test results, quality of life scores, and exercise tolerance after one year of follow-up [75]. However, their LVEF values of 50% and 47% were not typical for HFpEF, casting doubt on the effectiveness of the trial. In 2022, the CCM-HFpEF pilot study explored the use of CCM for the treatment of HFpEF [76]. The patients’ Kansas City Cardiomyopathy Questionnaire (KCCQ) composite score improved by 18.0 ± 16.6 points (*p* < 0.001) after 24 weeks of follow-up, and 93.6% of the patients did not experience any device- or procedure-related complications [76]. This trial provided a new strategy for the treatment of HFpEF, suggesting that CCM can significantly improve the health status of patients with HFpEF while ensuring their safety. Therefore, CCM may become another important nonpharmacological treatment for HFpEF.

Sinus rhythm is deemed necessary for effective treatment of CCM because the current CCM signal delivery algorithm requires sequential sensing of a *p* wave, followed by depolarizations at each ventricular lead [77]. It is noteworthy that most countries still consider AF a contraindication for CCM treatment. However, Röger et al. [78] found that CCM signal delivery is feasible in HF patients with permanent AF by sequential atrial-ventricular pacing, possibly due to the interpretation of the atrial pacing spike as a *p* wave by the CCM signal delivery algorithm. Additionally, the CCM-HFpEF pilot study [76] included almost half of the AF patients with HFpEF, revealing improvements in the KCCQ quality of life assessment. This suggests that CCM may offer encouraging potential benefits in improving the quality of life for HFpEF patients even when AF is present.

CCM treatment for patients with AF and HFpEF is still in the exploratory phase, and its actual clinical benefits are not yet clear. Some case reports and small sample studies have suggested that CCM may benefit patients with HFpEF; however, more robust evidence is necessary to support this claim. Therefore, routine CCM treatment for patients with AF and HFpEF is not currently recommended.

### Autonomic neuromodulation therapies (ANMTs)

The autonomic nervous system (ANS) includes the sympathetic and parasympathetic nervous systems. The sympathetic nervous system increases the heart rate, enhances myocardial contractility, and promotes cardiac conduction. The parasympathetic nervous system innervates the sinoatrial node and atrioventricular bundle and branches and produces effects opposite to those of the sympathetic nervous system [79]. Overactivation of the sympathetic nervous system causes the opening of L-type calcium channels, which leads to an increase in intracellular calcium ions. This enhances Na^+^-Ca^2+^ exchange and automaticity in myocardial cells and results in early depolarization, which can induce AF. Overstimulation of the parasympathetic nervous system causes acetylcholine to bind to muscarinic potassium channels and produce a hyperpolarization current, which markedly shortens the atrial effective refractory period, leading to the occurrence of AF [80]. The physiological effects of the sympathetic and parasympathetic nervous systems are mutually exclusive; however, the two systems can function synergistically to promote AF when both are overactivated.

**Fig. 3:**
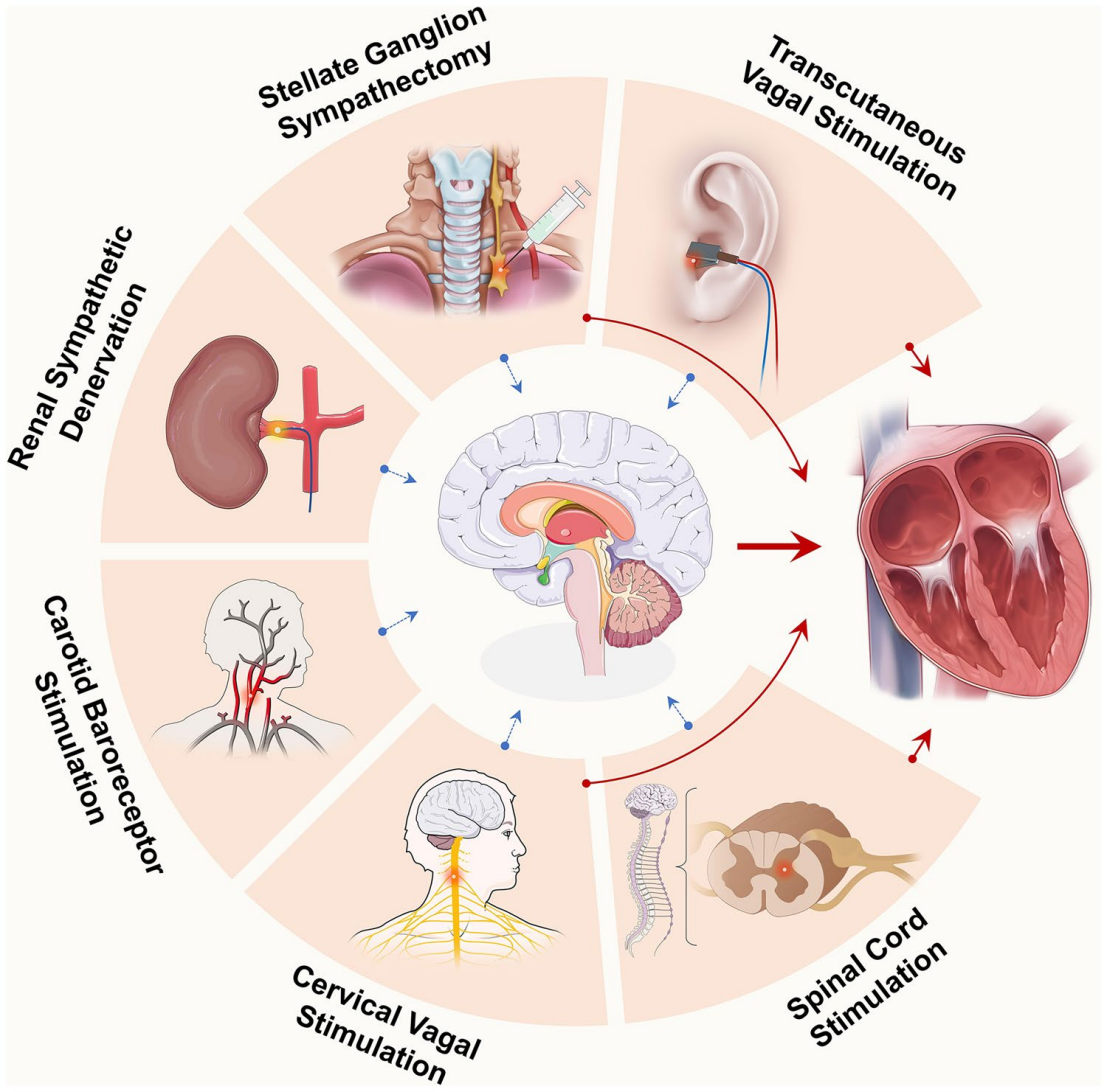
Autonomic neuromodulation therapies

ANMTs are emerging treatment options that can regulate the ANS through surgical intervention or device-based therapy to suppress the occurrence of AF [81]. ANMTs typically include ganglion plexus ablation, epicardial injection of botulinum toxin, VNS, stellate ganglion block, baroreceptor activation therapy, earlobe VNS, spinal cord stimulation, and renal denervation; among those, VNS has been clinically implemented (Fig. 3) [79, 82–84].

VNS is a closed-loop, self-powered system that typically includes a stimulator and an implanted electrode. The stimulator was placed in the left subclavian area, and the electrode end with three helical coils was wrapped around the vagus nerve in the left carotid sheath. The external programmable controller allows adjustment of the VNS by regulating the stimulation mode and parameters [85]. Continuous low-frequency stimulation of the vagus nerve through VNS releases acetylcholine, which binds to nicotinic acetylcholine receptors on tissue macrophages, inhibiting the release of inflammatory factors and reducing myocardial damage [86–88]. Furthermore, VNS promotes cardiac electrical stability by reducing the loss of connexin 43 and has a certain reversal effect on electrical remodeling [82]. VNS was initially developed to treat refractory epilepsy; however, it has proven to be effective in significantly shortening the duration of paroxysmal AF and improving myocardial fibrosis [89]. It is also a safe and feasible option for patients with postoperative AF [88]. VNS can reduce cardiac inflammation and fibrosis and improve cardiac diastolic function in patients with HFpEF and has been validated in Dahl salt-sensitive rat models [90]. Recent studies have shown that continuous VNS for more than 3 months may significantly improve the quality of life and reduce the level of tumor necrosis factor-α in patients with HFpEF [91].

ANMTs are novel technologies that remain in the early stages of exploration. Despite extensive research on VNS, there is still a paucity of reliable evidence regarding its safety and effectiveness in patients with AF and HFpEF. Considering its significant benefits for patients with AF and HFpEF, VNS may emerge as a new alternative device therapy for these patients in the future.

### Left ventricular expanders

Left ventricular expanders (LVEs) are spring-like devices implanted in the left ventricle to store elastic energy during cardiac contraction, releasing it during diastole. This process enhances left ventricular filling capacity, which is typically diminished in HFpEF [92].

The ImCardia and the CORolla transapical approach (CORolla TAA) devices are currently under development as two types of LVEs intended for implantation in the pericardium and endomyocardium, respectively. [92] ImCardia is an elastic, self-expanding device composed of a series of springs, with free lengths ranging from 35 to 46 mm, which are connected to attachment elements and screwed into the epimyocardium of the LV free wall 17 to 28 mm apart [93]. In animal models of diastolic dysfunction, ImCardia has been shown to be safe and effective in improving filling dynamics and enhancing cardiac contractility [94]. Furthermore, a prospective non-randomized study (NCT01347125) enrolled 19 HFpEF patients who underwent aortic valve replacement and were followed for 36 months. Although the intervention group exhibited no change in LVEF, reductions in left ventricular myocardial mass and left atrial area were observed. However, due to the complexities associated with implanting invasive devices, the trial had to be prematurely terminated.

CORolla TAA is a conical LVE composed of three elastic arms that can be implanted into the left ventricle through a transapical approach. Animal experiments have indicated a low incidence of adverse events associated with CORolla TAA implantation. Among 76 sheep models, one sheep experienced a significant decrease in LVEF, and two sheep developed mitral valve regurgitation. Active thrombi were detected in seven sheep, but all of them remained free of thrombosis after receiving antiplatelet therapy [95]. In an ongoing first-in-human clinical trial (NCT02499601), a 24-month follow-up is being conducted to assess the effectiveness and safety of CORolla TAA in 10 patients with HFpEF. One patient exhibited improvements in New York Heart Association (NYHA) functional class, KCCQ score, and a 6-min walk test at 6 months post-procedure. Additionally, at 12 months post-procedure, cardiac ultrasound indicated a reduction in left ventricular mass index (from 122 to 142 g/m^2^), left ventricular volume index (from 43 to 58 mL/m^2^), and left ventricular end-diastolic volume index (from 49 to 84 mL/m^2^). However, at 24 months post-procedure, the left ventricular mass index and left ventricular volume index increased to 130 g/m^2^ and 55 mL/m^2^, respectively, and the KCCQ score worsened to 44 points. Concerns about long-term cardiac function deterioration associated with CORolla TAA have been raised. This may be related to factors such as material fatigue, crack propagation, or stress corrosion cracking, necessitating further clinical trials to confirm the effectiveness and safety of CORolla TAA in HFpEF.

In short-term follow-up, LVEs have shown benefits for HFpEF patients, but their long-term effectiveness and safety remain uncertain. Currently, there is a lack of research evidence regarding LVEs in patients with AF and HFpEF. Furthermore, issues related to complications arising from LVE implantation, such as endomyocardial adhesions, valve damage, electrical conduction abnormalities, and potential impacts on pacemaker function, all require further investigation.

### Others

In addition to conventional medical treatments, device-based therapies for heart valve diseases appear to offer potential benefits to patients with AF and HFpEF. Transcatheter aortic valve replacement (TAVR) and transcatheter edge-to-edge repair (TEER) are two eagerly anticipated minimally invasive procedures for the treatment of heart valve diseases. Both TAVR and TEER have demonstrated their effectiveness and safety in managing heart valve diseases, especially in elderly individuals at high risk who may not be suitable candidates for traditional surgical procedures [96, 97].

In patients with severe aortic stenosis (AS) and HF, TAVR has demonstrated a lower in-hospital mortality rate than surgical aortic valve replacement [98]. A retrospective cohort study involving 66 patients with severe AS and HFpEF revealed that TAVR significantly reduced pulmonary artery systolic pressure and the peak aortic valve gradient and led to an improved NYHA functional class at 1 month post-procedure [99]. For individuals with both AS and HFpEF, TAVR emerges as a promising therapeutic option. Nevertheless, new-onset AF is among the postoperative complications associated with TAVR. According to findings from the SOURCE XT study, preexisting AF was prevalent in 35.6% of TAVR patients, with a 7.2% incidence of new-onset AF. AF is linked to higher rates of all-cause mortality, cardiac mortality, and bleeding events in TAVR patients [100]. Consequently, the addition of AF ablation surgery to TAVR may provide supplementary clinical benefits.

TEER has demonstrated promising prognostic improvements in patients with AF and HFpEF, particularly in cases of moderate to severe mitral regurgitation (MR). Several observational trials have reported significant efficacy of TEER in ameliorating symptoms and enhancing the quality of life among HFpEF patients, a substantial portion of whom also present with AF [101, 102]. Moreover, findings from substantial randomized controlled trials (RCTs), such as the COAPT study, suggest a notable trend toward reduced hospitalization due to HF and decreased all-cause mortality among HF patients with moderate to severe secondary MR who undergo mitral valve clip therapy [103]. Notably, although COAPT’s primary focus was on patients with HFrEF, it is worth highlighting that nearly one-sixth of the participants had an ejection fraction exceeding 40%, underscoring TEER’s potential in addressing HFpEF.

The tricuspid valve, while often overshadowed in clinical discussions, plays a crucial role in the context of HFpEF, as indicated by recent research [104, 105]. Secondary TR, stemming from left HF, arises due to elevated left ventricular filling pressures. This pressure increase triggers right ventricular overload, leading to dilation of both the right heart and the tricuspid annulus, culminating in TR [106, 107]. The presence of AF can exacerbate this by causing further dilation of the tricuspid annulus [108]. This creates a detrimental feedback loop in patients with concurrent AF and TR. Recent retrospective studies have underscored the potential of tricuspid TEER in ameliorating cardiac function and possibly reducing mortality rates in patients grappling with severe TR and HFpEF [109]. This emphasizes the pivotal role of the tricuspid valve in managing HFpEF patients. However, a gap exists in the form of large-scale RCTs assessing TEER’s utility in AF and HFpEF patients, especially those with varying TR severities. This underscores the pressing need for more research to formulate holistic guidelines for tricuspid valve interventions in this demographic.

TAVR, as a minimally invasive surgical approach, holds promise for patients with heart valve diseases and HFpEF. When coupled with AF, adjunctive ablation procedures may provide additional benefits. Furthermore, TEER, recognized as an efficient interventional therapy, may hold potential benefits for patients with both AF and HFpEF who experience MR or TR. Although large-scale RCTs targeting specific patient populations are currently lacking, further clinical trials will contribute to validating the effectiveness and safety of both approaches.

## Future directions and research

### The need for RCTs

Instrument-based therapies have resulted in significant benefits to patients; however, the issue of surgical complications cannot be ignored, and different devices have their own limitations [54, 110]. Currently, there is limited trial evidence for the majority of instrument-based therapies in patients with AF combined with HFpEF. Furthermore, the efficacy of these therapies remains uncertain, which questions their ability to improve long-term patient outcomes. Therefore, it is necessary to strictly understand and follow the indications and contraindications of instrument-based therapies and use constantly updated new therapeutic equipment to enable maximum therapeutic and diagnostic benefit to patients.

A rigorous evaluation of alternative treatments for AF and HFpEF is required to establish their safety, efficacy, and potential clinical benefits. Currently, there is a need for large-scale RCTs that investigate non-pharmacological treatment options, such as ablation therapy, CRT, CCM, VNS, LVEs, and others, in patients with AF and HFpEF. These trials should be designed to compare novel treatment modalities against standard pharmacological therapies or in combination with them to determine optimal therapeutic strategies for managing these conditions. Additionally, RCTs should evaluate the long-term safety and efficacy of these alternative treatments and assess their impact on patient-reported outcomes, such as symptom burden, functional capacity, and quality of life.

### Challenges in implementing new treatment modalities

The implementation of novel treatment modalities for AF and HFpEF introduces some challenges. Many new treatment options, particularly those involving advanced technologies or devices, may be associated with higher costs, limiting their accessibility for patients and healthcare systems [111]. Strategies to reduce costs and improve access to these treatments must be explored. Furthermore, the successful implementation of new treatment modalities requires adequate training and expertise among healthcare providers. This may involve the development of training programs, guidelines, and best practices to ensure proper execution and patient safety.

Identifying the most appropriate patient population for specific alternative treatments is crucial to optimize outcomes [112]. Researchers and clinicians must develop robust criteria for patient selection and account for factors such as disease severity, comorbidities, and patient preferences. The integration of new treatment options into clinical practice may face resistance from the medical community because of factors such as unfamiliarity, perceived risks, or concerns about the level of evidence supporting the new treatments. Continuous education, communication, and collaboration among healthcare professionals are essential for overcoming these barriers. The potential benefits of novel treatment approaches for AF and HFpEF can be more effectively realized after addressing these challenges, ultimately leading to improved patient care and outcomes.

## Conclusions

AF and HFpEF are two interrelated conditions that have garnered increasing attention owing to their rising prevalence and significant impact on healthcare systems worldwide. The complex pathophysiology of these twin diseases is not completely understood, and the limitations of current pharmacological treatments lead to the inadequate management of many patients. In recent years, alternative treatment modalities, such as ablation therapy, CRT, CCM, VNS, LVEs, and others, have emerged as promising approaches to address the unmet clinical needs of patients with AF and HFpEF. These novel interventions have the potential to revolutionize patient care and outcomes by providing more effective and targeted therapies.

However, several challenges must be addressed to fully realize the benefits of these innovative treatment options, including cost and accessibility, training and expertise, patient selection, and resistance from the medical community. Furthermore, rigorous evaluation using RCTs is essential to establish the safety, efficacy, and clinical utility of these alternative treatments for the management of AF and HFpEF. Overcoming these challenges and pursuing further research into novel therapeutic approaches can improve the care of patients with AF and HFpEF, ultimately enhancing their quality of life and reducing the burden on healthcare systems worldwide.

## Data Availability

No new data were generated or analyzed in support of this research.

## Abbreviations

AF: Atrial fibrillation
ANMTs: Autonomic neuromodulation therapies
ANS: Autonomic nervous system
AS: Aortic stenosis
BNP: B-type natriuretic peptide
BVP: Biventricular pacing
CCM: Cardiac contractility modulation
CRT: Cardiac resynchronization therapy
GDMT: Guideline-directed medical therapy
HBP: His bundle pacing
HF: Heart failure
HFpEF: Heart failure with preserved ejection fraction
HFrEF: Heart failure with reduced ejection fraction
KCCQ: Kansas City cardiomyopathy questionnaire
LBBB: Left bundle-branch block
LBBP: Left bundle-branch pacing
LVEF: Left ventricular ejection fraction
LVEs: Left ventricular expanders
MR: Mitral regurgitation
NYHA: New York Heart Association
PFA: Pulsed field ablation
PVI: Pulmonary vein isolation
RCTs: Randomized controlled trials
RFA: Radiofrequency ablation
TAA: Transapical approach
TAVR: Transcatheter aortic valve replacement
TEER: Transcatheter edge-to-edge repair
TR: Tricuspid regurgitation
VNS: Vagus nerve stimulation

## Code availability

Figures 2 and 3 were created using the image bank of Servier Medical Art (http://smart.servier.com/), licensed under a Creative Commons Attribution 3.0 Unported License (https://creativecommons.org/licenses/by/3.0/).
